# Impact of the age of onset and duration of schizophrenia on the quality of treatment adherence

**DOI:** 10.1192/j.eurpsy.2025.2251

**Published:** 2025-08-26

**Authors:** A. Rami

**Affiliations:** Razi Hospital, Manouba, Tunisia

## Abstract

**Introduction:**

Schizophrenia is a chronic, frequent, and disabling psychiatric condition. The prognosis is more severe in the absence of treatment.

**Objectives:**

The aims of our study were to evaluate the quality of treatment adherence and the Impact of the age of onset and duration of schizophrenia on the quality of treatment adherence and to assess the implication of these factors as predictors of poor adherence.

**Methods:**

We conducted a cross-sectional and analytical study. We recruited 150 patients with schizophrenia treated at Razi Hospital of Manouba, divided into 113 patients with good adherence compared to 37 patients with poor adherence. We used the Medical Adherence Report Scale (MARS) to assess the quality of therapeutic adherence.

**Results:**

The average age of onset of the illness was 22.91 ± 4.6 years, with extremes ranging from 13 to 36 years.

The average duration of the illness in the patients in our series was 17 years, with extremes ranging from two to 42 years.

The average duration of untreated psychosis was two years, with a median of 12 months and extremes ranging from one month to 20 years.

A statistically significant association was found between the duration of untreated psychosis and the quality of treatment adherence (p=0.003).

Neither the age of onset of the illness nor its duration had any influence on the quality of patient adherence.

**Conclusions:**

To prevent poor treatment adherence, a systematic screening for predictive factors and adequate management of schizophrenia would be imperative.

**Disclosure of Interest:**

None Declared

